# Bridging Borders for Health: The Vital Role of Regional Cooperation in Infectious Disease Control and Mitigation of Health Emergencies; A Response to the Recent Commentaries

**DOI:** 10.34172/ijhpm.2023.8200

**Published:** 2023-08-06

**Authors:** Anna Durrance-Bagale, Li Yang Hsu, Natasha Howard

**Affiliations:** ^1^Department of Global Health & Development, London School of Hygiene & Tropical Medicine, London, UK; ^2^Saw Swee Hock School of Public Health, National University of Singapore and National University Health System, Singapore, Singapore

## Background

 The COVID-19 pandemic underlined the importance of effective regional collaboration to control infectious diseases. In December 2021, we published a scoping review in this journal examining research on how to operationalise regional bodies to effectively address potential infectious disease threats.^1^ Key enablers included clear understanding of the regional context, sufficient budgeting, addressing cultural/language issues, staffing capacity, and governmental priorities. Initial engagement among institutional bodies involved in design, implementation, monitoring, or evaluation of such collaborations is essential, as are a transparent governance structure with clear responsibilities and secure long-term funding.

## Progress

 In 2022, Standley and Sorrell^2^ and Teerawattananon et al^3^ published two constructive commentaries on our work. Standley and Sorrell identified our lack of emphasis on ‘regional networks established by an external entity or donor’ (eg, US-CDC Disease Detection centres), noting dependency on external funding as a barrier to successful regional engagement. We agree, considering how these externally funded networks often function alongside national governance structures and priorities, and often — unintentionally or intentionally — exclude them. Thus, the influences of external funding on regional governance and technical bodies are an important area for further research. Teerawattananon and colleagues^3^ discussed issues around establishing an Association of Southeast Asian Nations (ASEAN) regional disease control body and initiation of the ASEAN Center for Public Health Emergencies and Emerging Diseases (ACPHEED) to prepare for the next pandemic. They outlined recommendations for fostering regional cooperation that reflect our findings, including identifying innovative financing mechanisms, applying a One Health approach, and involving private sector stakeholders. Teerawattananon and colleagues^3^ agreed with Standley and Sorrell^2^ that external funders, including private-sector funders, may play a key role in any future initiative.

 Building on our review findings and to help inform ACPHEED operationalisation, we interviewed 23 senior-level experts in regional organisations or networks globally, on their experiences with operationalising such bodies.^4^ Interviewee opinions tallied with review findings, and those of Standley and Sorrell^2^ and Teerawattananon et al,^3^ highlighting governance and diplomacy, financing, capacity-building, stakeholder engagement, and multilateral agreements as key to encouraging effective operationalisation. Interviewees further emphasised the importance of a One Health approach,^5^ the need to clarify how effective regional collaborations are — including through routine monitoring and evaluation, and diverse financing options — including potential private-sector involvement.

## Where Next…

 ACPHEED establishment was agreed at the 15th ASEAN Health Ministers Meeting, in May 2022, with a November 2022 launch, and Japan committing US$ 50 million of financial support.^6,7^ In April 2023, Japan reaffirmed its commitment of US$50 million to develop ACPHEED, citing this as a step toward addressing global public health risks and strengthening cooperation within ASEAN.^8^ These steps are encouraging. Lessons from the COVID-19 pandemic on the importance of sharing data, working with both proximal and distal countries, and effective resource pooling, must be incorporated for networks like ACPHEED to be effective. A notable aspect of ACPHEED progress is the decision to devolve functionally into a tripartite structure with secretariat in Bangkok and detection, response, and risk management based in Vietnam, Indonesia, and Thailand. Further research on operationalising regional disease control cooperation and ongoing learning from ACPHEED’s development and governance will help ensure we are better prepared for the next pandemic.

## Acknowledgements

 We thank the original review team^1^ and authors of the two commentaries^2,3^ for their relevant contributions to this discussion.

## Ethical issues

 Not applicable.

## Competing interests

 Authors declare that they have no competing interests.
